# Predictive Ability of an Objective and Time-Saving Blastocyst Scoring Model on Live Birth

**DOI:** 10.3390/biomedicines13071734

**Published:** 2025-07-15

**Authors:** Bing-Xin Ma, Feng Zhou, Guang-Nian Zhao, Lei Jin, Bo Huang

**Affiliations:** 1 Reproductive Medicine Center, Tongji Hospital, Tongji Medical College, Huazhong University of Science and Technology, Wuhan 430074, China; mabx@tjh.tjmu.edu.cn; 2 Department of Infection Control, Renmin Hospital of Wuhan University, Wuhan 430060, China; zhoufeng12@wust.edu.cn; 3 Department of Obstetrics and Gynecology, National Clinical Research Center for Obstetrics and Gynecology, Tongji Hospital, Tongji Medical College, Huazhong University of Science and Technology, Wuhan 430074, China; zhaogn@tjh.tjmu.edu.cn

**Keywords:** iDAScore, time-lapse incubator, single vitrified–thawed blastocyst transfer, clinical outcome, neonatal outcome

## Abstract

**Objectives:** With the development of artificial intelligence technology in medicine, an intelligent deep learning-based embryo scoring system (iDAScore) has been developed on full-time lapse sequences of embryos. It automatically ranks embryos according to the likelihood of achieving a fetal heartbeat with no manual input from embryologists. To ensure its performance, external validation studies should be performed at multiple clinics. **Methods**: A total of 6291 single vitrified–thawed blastocyst transfer cycles from 2018 to 2021 at the Reproductive Medicine Center, Tongji Hospital, Tongji Medical College, Huazhong University of Science and Technology were retrospectively analyzed by the iDAScore model. Patients with two or more blastocysts transferred and blastocysts that were not cultured in a time-lapse incubator were excluded. Blastocysts were divided into four comparably sized groups by first sorting their iDAScore values in ascending order and then compared with the clinical, perinatal, and neonatal outcomes. **Results:** Our results showed that clinical pregnancy, miscarriage, and live birth significantly correlated with iDAScore (*p* < 0.001). For perinatal and neonatal outcomes, no significant difference was shown in four iDAScore groups, except sex ratio. Uni- and multivariable logistic regressions showed that iDAScore was significantly positively correlated with live birth rate (*p* < 0.05). **Conclusions**: In conclusion, the objective ranking can prioritize embryos reliably and rapidly for transfer, which could allow embryologists more time for processes requiring hands-on procedures.

## 1. Introduction

According to previous studies, elective single blastocyst transfer (eSBT) is considered the most efficient way to lower the chance of multiple pregnancies following in vitro fertilization (IVF) and reduces adverse neonatal and obstetric outcomes [1,2]. Selection of the most viable blastocyst for transfer is essential for a successful outcome. Traditionally, transferred blastocysts are assessed using the Gardner blastocyst grading system [3]. According to this system, the developmental stage, the size, and the quality of the inner cell mass (ICM) and trophectoderm (TE) are evaluated by embryologists. Although the clinical pregnancy rate of blastocysts selected by this method has been greatly improved, it is prone to the subjectivity of the embryologists and is very dependent on the embryologist’s experience [4]. Therefore, objective indicators and better performance are required to help embryologists to prioritize blastocysts with the highest likelihood of a positive clinical outcome.

**Figure 1:**
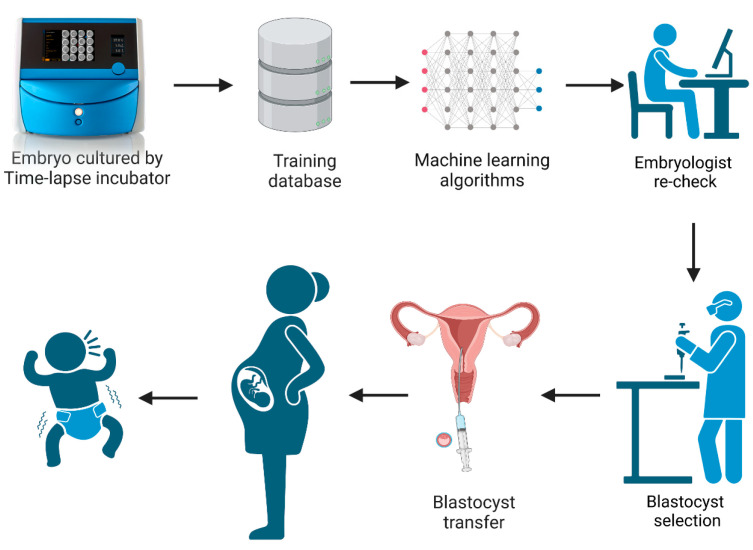
The workflow diagram of iDAScore. (Created with BioRender.com).

In recent years, there have been many studies documenting the application and usage of time-lapse culture systems in IVF [5,6,7,8]. A time-lapse system monitors embryo development without the need to remove the culture dish from the incubator. This undisturbed culture offers an optimal constant micro-environment for embryos during the whole culture period while allowing for the continuous acquisition of dynamic images. This improves embryo evaluation by having the full development history of the embryo, including dynamic morphology, morphokinetics, and the identification of abnormal cell divisions [9,10]. However, the timings of cell division and abnormal behaviors still need to be observed and annotated by embryologists, which is time-consuming. With the development and wide application of artificial intelligence technology, the iDAScore (Vitrolife A/S, Gothenburg, Sweden) embryo selection model was created based on the IVY deep learning model by training 115,832 embryos and 14,644 known implantation embryos from 18 reproductive medical centers [11,12,13]. It is an optional software which can be directly integrated in the EmbryoScope Plus system (Vitrolife A/S, Gothenburg, Sweden). The system provides a score between 1–9.9 for all patient embryos, without any manipulation and intervention by embryologists. The workflow diagram of iDAScore is presented in Figure 1. Ueno et al. reported that the blastocysts selected by iDAScore perform as well or even better than with the traditional Gardner criteria and annotation-dependent ranking systems [14]. Although the performance of iDAScore has previously been validated, it has not yet been externally validated in a large cohort study [11,12]. As a result, the generalizability of iDAScore should be assessed in a larger number of reproductive clinics.

In this study, we aimed to determine if iDAScore values reliably reflect clinical pregnancy and live birth (LB) in vitrified–thawed eSBT cycles at the Reproductive Medicine Center, Tongji Hospital, Tongji Medical College, Huazhong University of Science and Technology. In addition, the perinatal and neonatal outcomes according to iDAScore values were analyzed.

## 2. Materials and Methods

### 2.1. Study Design

**Figure 2:**
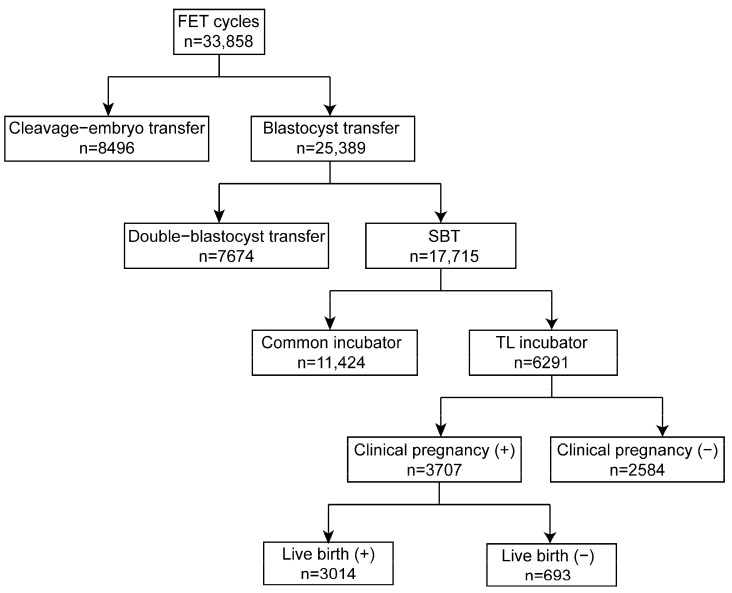
Schematic presentation of the study design. FET: frozen embryo transfer; eSBT: elective single blastocyst transfer.

The retrospective cohort study involved 33,858 cycles conducted from 2018 to 2021 at the Reproductive Medicine Center, Tongji Hospital, Tongji Medical College, Huazhong University of Science and Technology, Wuhan, Hubei, China. Of these, 8469 cycles were cleavage embryo transfers, while 25,389 cycles were blastocyst transfers. Among the patients, 7674 had double blastocyst transfers, while 17,715 were eSBT cycles. In the present study, 6291 blastocysts cultured in an EmbryoScope Plus (Vitrolife A/S, Gothenburg, Sweden) incubator were retrospectively analyzed using the iDAScore model. The study design is shown in Figure 2. Every patient signed an informed consent form. The study complied with the Declaration of Helsinki for Human Subjects in Medical Research and the Board of Institutional Review (No. 2019s097) approval was given by the Ethical Committee of Reproductive Medicine Center, Tongji Hospital, Tongji Medicine College, Huazhong University of Science and Technology.

### 2.2. Clinical Protocol

Controlled ovarian stimulation (COS) was performed in accordance with our previous studies [5]. Transvaginal ultrasonography was used to closely follow patients during COS. The leading follicle(s) were given human chorionic gonadotropin (HCG) when they measured >18 mm. Oocyte retrieval under ultrasound guidance was performed 36 h after HCG injection.

### 2.3. Laboratory Protocol

The procedure for collecting semen/cumulus–oocyte complexes (COCs), insemination, and embryo culture was performed as previously described [5]. The semen samples were optimized using density gradient centrifugation. The World Health Organization fifth edition guidelines were used to assess the concentration, motility, and morphology of sperm. The fertilization method was selected according to sperm density and quality. The COCs were inseminated with 10,000 motile spermatozoa each in the IVF cycle. The COCs were denuded two hours after retrieval during intracytoplasmic sperm injection (ICSI) cycles, and sperm was injected four hours later. The zygotes produced were then transferred to G1 Plus (Vitrolife, Gothenburg, Sweden) and cultivated using a time-lapse incubation system. Every 10 min, each embryo was photographed by the system. Pronuclei were examined 16 to 18 h after insemination. On the third day, the culture medium was changed to G2 Plus (Vitrolife, Gothenburg, Sweden). On the fifth and sixth day, the blastocysts better than 3BC (Gardner criteria) were cryopreserved for future use. In rare cases, the embryo was cultured until the seventh day for vitrification.

### 2.4. FET Protocols

Transvaginal ultrasound examination was used to gauge endometrial thickness, follicle development, and ovulation in natural cycles. Blood progesterone levels were measured at cycle days 10 to 12. Three days after ovulation, blastocysts were thawed and transferred. The order of blastocyst thawing were according to the Gardner score before cryopreservation. Progesterone was administered intramuscularly for luteal support beginning one day after ovulation.

For hormone replacement therapy cycles, oral estradiol (Progynova; Bayer; Leverkusen, Germany) was administered in doses of 2 mg/day, 4 mg/day, and 6 mg/day from days 1 to 4, 5 to 8, and 9 to 12, respectively. If the endometrium thickness reached 8.0 mm on the 13th day, 40 mg progesterone was given intramuscularly for three days. After three days of progesterone injection, the blastocyst was thawed and transplanted.

Transvaginal ultrasonography was used to detect fetal heart activity in the uterus four weeks following the transfer to confirm clinical pregnancy. Loss of the gestational sac or fetal heartbeat within 20 weeks of a clinical pregnancy diagnosis was defined as miscarriage.

### 2.5. iDAScore Model

Using Python 3.6.5 and TensorFlow v2.0, an iDAScore embryo scoring model v1.0 was developed for the binary classification of positive and negative fetal hearts among 98,583 embryos in the training dataset. Additionally, the training process was carried out with the assistance of two Nvidia Quadro RTX8000 GPUs11.

The model received an input comprising 128 frames, each sampled at an interval of one hour, captured on a single focal plane with a resolution of 256 × 256 pixels. The sequence began at 12 h post-insemination (hpi) and extended to cover the period from 12 to 140 hpi. Then, the sampled data sequence underwent several preprocessing steps, including temporal coherent random cutout, rescaling between 90–110%, horizontal and vertical translations up to 10%, brightness adjustments, horizontal flipping, etc. For both of these outputs, the focal loss function was employed, with a gamma value of 2.0 and an alpha of 0.511. Upon the completion of training, the fetal hearts output generated by the model was rescaled from the range of [0, 1] to [1.0, 9.9].

In the present study, data from the included blastocysts were scored by the iDAScore model retrospectively. Blastocysts were divided into four comparably sized groups by first sorting their iDAScore values in ascending order. The following intervals were used: 1.0–8.0, 8.1–8.9, 9.0–9.3, and 9.4–9.9. However, since a larger proportion of the selected blastocysts had a score of 9.3, the 9.0–9.3 group contained more blastocysts than the 9.4–9.9 score group.

### 2.6. Statistical Analysis

Continuous variables with normal distributions were expressed as mean ± SD. Categorical variables were expressed as number and percentage (%).

For data with a normal distribution, one-way analysis of variance (ANOVA) was applied for multiple comparisons, with Bonferroni post hoc analysis for data meeting homogeneity of variance or with Tamhane’s T2 analysis for data demonstrating heteroscedasticity.

For data with a skewed distribution, the Kruskal–Wallis test was followed by the Dunn’s test for pairwise comparisons. The chi-square test or Fisher’s exact test with Bonferroni correction was used to compare categorical variables between groups, and the Cochran–Armitage test was used to detect trends in rates across groups of nominal variables.

Multivariable logistic regression was applied to evaluate the association between the iDAScores and LB, and the odds ratios (ORs) were calculated.

All statistical tests were two-sided, and *p* values less than 0.05 were considered statistically significant. All analyses were conducted in SPSS Statistics (version 23.0, IBM, Armonk, NY, USA) and R-3.6.3 (R Foundation for Statistical Computing, Vienna, Austria).

## 3. Results

### 3.1. Patient Characteristics in Different iDAScore Groups

The schematic presentation of the study design is shown in Figure 2. In the present study, 6291 blastocysts were involved and divided into equal-sized groups according to iDAScore quartiles. As shown in Table 1, the average maternal age was 31.5. The age difference between groups 1.0–8.0 and 8.1–8.9 was not statistically significant, and neither was the difference between groups 9.0–9.3 and 9.4–9.9. The other paired comparisons were statistically significant (*p* < 0.001). Higher iDAScores had a shorter cryopreservation duration. Significant differences were observed in all groups except between iDAScore groups 9.0–9.3 and 9.4–9.9 (*p* < 0.001). As such, high-quality blastocysts were more likely to be transferred. Endometrial thickness was similar for all groups, with an average thickness of 9.4, with no significant difference observed. Regimens of endometrial preparations for frozen embryo transfer were similar in all groups (*p* > 0.05). Embryos that reached the blastocyst stage by day 5 had higher iDAScores than those reaching the blastocyst stage on day 6 or 7. Significant differences were shown in all groups (*p* < 0.001).

### 3.2. Clinical Outcomes of Blastocysts in Different iDAScore Groups

**Figure 3:**
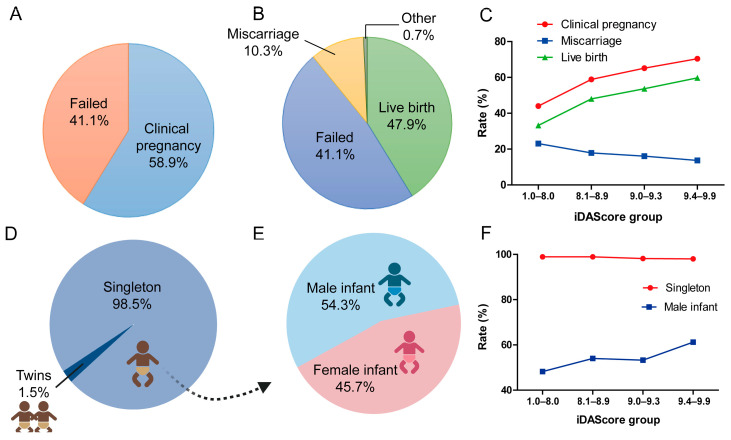
Clinical outcomes of blastocysts in different iDAScore groups. (**A**) Clinical pregnancy rate of all cycles. (**B**) Live birth rate of all cycles. (**C**) Clinical pregnancy, miscarriage, and live birth rates in different iDAScore groups. (**D**) Singleton rate of all live births. (**E**) Male infant rate of all live births. (**F**) Singleton and male infant rates in different iDAScore groups. *p* < 0.001 for clinical pregnancy, miscarriage, and live birth rates in different iDAScore groups; values were calculated using the Cochrane–Armitage trend test. *p* > 0.05 for singleton rate, *p* < 0.001 for male infant rate; values were calculated by chi-square analysis for singleton and male infant rates. (Created with BioRender.com).

Among 6291 eSBT cycles, 3707 (58.9%) cycles achieved at least 1 positive fetal heartbeat and were considered as a clinical pregnancy (Figure 3A). Of the 6291 cycles, 3014 (47.9%) resulted in LB, 645 (17.4%) in miscarriage, 24 (0.3%) in ectopic pregnancies, and 5 in stillbirths (0.1%) (Figure 3B). Figure 3C shows the clinical outcomes in different iDAScore groups. The clinical pregnancy rate significantly increased from 44.0% to 70.4% (*p* < 0.001). The LB rate significantly increased from 33.2% to 59.8% for the highest iDAScore group (*p* < 0.001). The miscarriage rate significantly decreased with the increase in iDAScore, from 23.1% to 13.7% (*p* < 0.001). 

### 3.3. Perinatal and Neonatal Outcomes of Blastocysts in Different iDAScore Groups

For perinatal outcome, 98.5% of cycles were singleton births, while 1.5% were twins (Figure 3D). The ratio of male infant of all cycles was 54.3% (*p* < 0.001, Figure 3E). Furthermore, infant sex differed significantly for singletons between the different iDAScore groups (*p* < 0.001, Figure 3F). In the high iDAScore group (9.4–9.9), more male infants were born than female infants (61.2% vs. 38.8%), whereas in 1.0–8.0 iDAScore group, fewer male infants were born than female infants (48.2% vs. 51.8%).

Other perinatal outcomes were analyzed in both singletons (Table 2) and twins (Supplementary Table S1). Gestational age and premature birth rate did not differ significantly between iDAScore quartiles (*p* > 0.05, Table 2). No significant difference was shown in type and rate of pregnancy complication (*p* > 0.05). For newborn babies, birth weights were statistically similar across all groups (*p* > 0.05).

The birth defects for boy and girl infants and single/twin infants are reported in Supplementary Table S2. No significant difference was found between the iDAScore groups for the different types of birth defect (*p* > 0.05).

### 3.4. Uni- and Multivariable Logistic Regression Analysis for LB

Table 3 shows the results of uni- and multivariable logistic regression analysis for LB. The multivariable logistic regression was adjusted for maternal age, endometrial thickness, and length of incubation. The iDAScore was significantly correlated with a positive LB probability in both the uni- and multivariable logistic regression.

## 4. Discussion

Over the years several time-lapse embryo selection models have been developed. Initially, these models were mostly based on morphokinetic parameters [15,16,17,18], while more recent models are based on deep learning [13,19]. However, common to all model types is that it is essential that the model is validated on an external dataset that has not been part of the model validation [20,21,22,23,24]. Ultimately, the models should also be tested in randomized prospective studies.

At present, only a few studies have investigated the clinical application of iDAScore. Ezoe et al. analyzed the relationship between iDAScore and morphokinetics and morphological scores and found that iDAScore was substantially associated with preimplantation embryo morphology and morphokinetics, particularly during compaction and blastulation [25].

In this study, 6291 cycles of single, vitrified blastocyst transfers were included and evaluated. We found that the clinical pregnancy rate and the LB rate significantly increased as the iDAScore increased (*p* < 0.001). Furthermore, the miscarriage rate significantly decreased as the iDAScore increased (*p* < 0.001). In addition, iDAScore was significantly correlated with a positive LB in a multivariable logistic regression analysis (aOR: 1.200, 95%; CI: 1.148–1.253, *p* < 0.05). Therefore, we conclude that iDAScore is a validated and reliable predictor of the likelihood of a clinical pregnancy, miscarriage, or live birth in our clinical setting. The significant correlations between iDAScore and LB are in agreement with previous studies [14]. Their study found that iDAScore significantly correlated with LB (aOR: 1.535, 95%; CI: 1.358–1.736, *p* < 0.05) in 3010 single embryo transfers. They also reported that iDAScore outperformed previously established methods for such an evaluation or annotation-dependent ranking systems [12]. After single vitrified blastocyst transfer, iDAScore may be used reliably to identify blastocysts with the highest chance of achieving live birth, especially for younger patients. Kato et al. also found that iDAScore, KIDScore Day 5, and Gardner criteria were significantly correlated with euploidy for blastocyst assessment [26]. However, the accuracy of ploidy status was not high enough for diagnostic use and should, thus, mainly be used for biopsy prioritization.

Our data showed a significant association between miscarriage rate and iDAScore. Thus, the miscarriage rate increased from 13.7% for a high iDAScore to 23.1% for a low iDAScore. The study by Ueno et al. also found a significant increase from 26.0% to 41.0% from a high iDAScore to a low iDAScore [14]. The higher absolute values observed in their study were probably due to the older patient cohort, with an average age of 39.3 years compared to an average age of 31.5 years in our study.

For perinatal and neonatal outcomes, we showed that iDAScore did not correlate with gestational age, pregnancy complication, birth weight, or any birth defect. This agrees with the study by Ueno et al., where they also showed that iDAScore did not correlate with neonatal outcomes [14]. For the singleton infant sex, we found an overall rate of 54.3% males. With regards to iDAScore we found that the low-scoring group had 48.2% males, which significantly increased to 61.2% males for the high-scoring group. In the study by Ueno et al. they found a non-significant increase from 43.7% for the low iDAScore group to 52.2% males for the high iDAScore group. Thus, both studies indicate that that there is a correlation between blastocyst quality and infant sex. Notably, skewed sex ratio has been an issue of great concern in the field of IVF since 1991 [27]. In recent years, Mao et al. reported that the secondary sex ratio of ART babies in the single blastocyte FET cycle was 1.24, and the sex ratio was associated with blastocyst quality [28]. Lou et al., and Cai et al., reported similar results [29,30]. A single high-quality blastocyst has a higher chance of resulting in a male infant than a female infant. In particular, trophectoderm grade was associated with a significantly higher sex ratio. It is reported that the skewed sex ratio is likely due to the faster cleavage rate in male blastocytes [31,32]. In fact, female embryos were reported to consume significantly more glucose than male ones [33]. Thus, more energy was needed to inactivate the second X chromosome, which might come at the cost of a slower mitotic rate [34]. Likewise, it is proposed that precocious X-chromosome inactivation and a reduction in trophectoderm cell quantity is more likely to appear in female blastocysts [35,36]. This implies that there is a chance that the sex ratio will move toward more male offspring in situations when blastocysts are chosen based on trophectoderm grade or growth. This correlation has also been found in several other studies based on morphology. For example, one study found that 72% of the blastocysts with the highest morphologic scores (Gardner Criteria, 5AA, and 6AA) were found to be male, compared with only 40% of grade 3 embryos [37]. Similarly, another study found that 62.5% of the transferred blastocysts with excellent morphology were male, while 50.8% of the transferred blastocysts with poor morphology were male [38]. It should also be noted that the above studies were retrospective where iDAScore was not used for selection. Thus, we propose that the impact of using any deep learning embryo selection model should be studied in a prospective randomized setting where the model is used for selection.

Although we cannot explain what iDAScore or similar deep learning embryo evaluation models have learned during training, the ultimate aim must be the prediction of the likelihood of having a healthy baby. This investigation found that iDAScore significantly positively correlated with LB rate and significantly negatively correlated with the miscarriage rate. In addition, there were no indications of a correlation with negative perinatal or neonatal characteristics. Overall, this external study supports that iDAScore can generalize to new clinics and safely facilitate the selection of embryos with the aim of increasing LB rates.

The main limitation of this study is that only vitrified–thawed blastocyst transfer cycles were included, while the fresh cycles and cleavage-stage embryos were not evaluated. Additionally, we only conducted a preliminary grouping based on the iDAScore values and did not make more detailed divisions of blastocysts according to other factors, such as maternal age. In future studies, we will need more data to conduct a more comprehensive and detailed assessment of the effectiveness of iDAScore.

## 5. Conclusions

The results of the present study show that iDAScore correlates with clinical pregnancy, miscarriage, and live birth in a clinic where external data have not been used for training the model. The model provides a rapid and reliable ranking of embryos, which may remove subjectivity of evaluation and optimize the use of clinical resources by saving time. No significant difference was observed in perinatal and neonatal outcomes, except for sex ratio. Research should continue in a real clinical setting to examine the utility of iDAScore for prioritizing the transfer of blastocysts in a prospective study.

## Figures and Tables

**Table 1:** Patient and cycle characteristics in different iDAScore groups.

iDAScore Group	All	1.0–8.0	8.1–8.9	9.0–9.3	9.4–9.9	*p* Value
Cycles, *n*	6291	1683	1556	1909	1143	/
Maternal age, mean ± SD, year	31.5 ± 4.2	31.9 ± 4.5	31.8 ± 4.1	31.2 ± 4.0	31.0 ± 4.2	<0.001 ^a^
Cryopreservation duration, median (IQR), day	66 (31–146)	86 (36–186)	67 (31–140)	62 (30–127)	60 (29–116)	<0.001 ^b^
Endometrial thickness	9.4 ± 1.5	9.3 ± 1.5	9.4 ± 1.6	9.4 ± 1.5	9.4 ± 1.4	0.078 ^c^
Regimen of endometrial preparation for frozen embryo transfer, n (%)						0.208 ^d^
Natural cycle	248 (4.0%)	66 (3.9%)	75 (4.8%)	68 (3.6%)	39 (3.4%)	
Programmed cycle	5922 (94.1%)	1583 (94.1%)	1459 (93.8%)	1804 (94.5%)	1076 (94.1%)	
Others	121 (1.9%)	34 (2.0%)	22 (1.4%)	37 (1.9%)	28 (2.5%)	
Length of incubation						<0.001 ^e^
Day 5	4706 (74.8%)	440 (26.1%)	1218 (78.3%)	1905 (99.8%)	1143 (100.0%)	
Day 6	1562 (24.8%)	1220 (72.5%)	338 (21.7%)	4 (0.2%)	0 (0.0%)	
Day 7	23 (0.4%)	23 (1.4%)	0 (0.0%)	0 (0.0%)	0 (0.0%)	

IQR: interquartile range; SD: standard deviation. ^a^ *p* value was calculated by an ANOVA test with Bonferroni correction for maternal age. Significant difference was shown in all groups except for comparison between iDAScore group 1.0–8.0 and iDAScore group 8.1–8.9, and between iDAScore group 9.0–9.3 and iDAScore group 9.4–9.9. ^b^ *p* value was calculated by the Kruskal–Wallis test for freezing time. Significant differences were shown in all groups except between iDAScore group 9.0–9.3 and iDAScore group 9.4–9.9. ^c^ *p* value was calculated by an ANOVA test with Bonferroni post hoc analysis for endometrial thickness. ^d^ *p* value was calculated by a chi-square analysis for different regimens. ^e^ *p* value was calculated by a chi-square test with Bonferroni analysis for length of incubation.

**Table 2:** Perinatal and neonatal outcomes for singleton pregnancy in different iDAScore groups.

iDAScore Group	All	1.0–8.0	8.1–8.9	9.0–9.3	9.4–9.9	*p* Value
Live birth, *n*	2968	552	739	1007	670	
Gestational age, mean ± SD, weeks	38.2 ± 1.6	38.2 ± 1.5	38.2 ± 1.7	38.3 ± 1.6	38.3 ± 1.6	0.707 ^a^
Early preterm birth (<37 weeks), *n* (%)	305 (10.3%)	63 (11.4%)	73 (9.9%)	100 (9.9%)	69 (10.3%)	0.794 ^b^
Very early preterm birth (<32 weeks), *n* (%)	23 (0.8%)	0 (0.0%)	9 (1.2%)	8 (0.8%)	6 (0.9%)	0.097 ^b^
Types of pregnancy complication, *n* (%)						0.645 ^c^
Gestational hypertension, *n* (%)	60 (2.0%)	10 (1.8%)	20 (2.7%)	18 (1.8%)	12 (1.8%)	
Gestational diabetes, *n* (%)	61 (2.0%)	8 (1.4%)	17 (2.2%)	20 (2.0%)	16 (2.3%)	
Pre-eclampsia, *n* (%)	6 (0.2%)	2 (0.4%)	3 (0.4%)	1 (0.1%)	0 (0.0%)	
Placenta previa, *n* (%)	33 (1.1%)	5 (0.9%)	7 (0.9%)	13 (1.3%)	8 (1.2%)	
Premature rupture of membranes, *n* (%)	43 (1.4%)	6 (1.1%)	9 (1.2%)	16 (1.6%)	12 (1.8%)	
Birth weight, *n* (%)						0.063 ^d^
<1500 g	16 (0.5%)	0 (0.0%)	10 (1.4%)	5 (0.5%)	1 (0.0%)	
1500–2499 g	144 (4.9%)	32 (5.8%)	36 (4.9%)	48 (4.8%)	28 (4.2%)	
2500–3999 g	2602 (87.7%)	484 (87.7%)	644 (87.1%)	883 (87.7%)	591 (88.2%)	
≥4000 g	206 (6.9%)	36 (6.5%)	49 (6.6%)	71 (7.0%)	50 (7.5%)	

SD: standard deviation. ^a^ *p* value was calculated by an ANOVA test with Bonferroni correction for gestational age. ^b^ *p* value was calculated by the Cochrane–Armitage trend test for early preterm birth (<37 weeks) and very early preterm birth (<32 weeks). ^c^ *p* value was calculated by chi-square analysis for different types of pregnancy complication. ^d^ *p* value was calculated by chi-square analysis for birth weight.

**Table 3:** Uni- and multivariable logistic regression analysis for live births.

	Univariable Analysis			Multivariable Analysis		
	Odds ratio	95 CI%	*p* value	Odds ratio	95 CI%	*p* value
iDAScore	1.285	1.239–1.333	<0.001	1.200	1.148–1.253	<0.001

## Data Availability

The datasets used and/or analyzed during the current study are available from the corresponding author on reasonable request.

## Supplementary Materials

The following supporting information can be downloaded at: https://www.mdpi.com/article/10.3390/biomedicines13071734/s1, Table S1: Perinatal and Neonatal outcomes for monozygotic twins pregnancy in different iDAScore groups; Table S2: Birth defect in different iDAScore groups.

## Abbreviations

The following abbreviations are used in this manuscript:

eSBT	Elective single blastocyst transfer
ICM	Inner cell mass
TE	Trophectoderm
COS	Controlled ovarian stimulation
HCG	Human chorionic gonadotropin
COCs	Cumulus–oocyte complexes
FET	Frozen embryo transfer
IVF	In vitro fertilization
ICSI	Intracytoplasmic sperm injection
LB	Live birth
OR	Odds ratio
IQR	Interquartile range

## References

[1] Kim H.J., Park J.K., Eum J.H., Song H., Lee W.S., Lyu S.W. (2021). Embryo Selection Based on Morphological Parameters in a Single Vitrified-Warmed Blastocyst Transfer Cycle. Reprod. Sci..

[2] Lin J., Zhao J., Hao G., Tan J., Pan Y., Wang Z., Jiang Q., Xu N., Shi Y. (2020). Maternal and Neonatal Complications After Natural vs. Hormone Replacement Therapy Cycle Regimen for Frozen Single Blastocyst Transfer. Front. Med..

[3] Gardner D.K., Lane M., Stevens J., Schlenker T., Schoolcraft W.B. (2000). Blastocyst score affects implantation and pregnancy outcome: Towards a single blastocyst transfer. Fertil. Steril..

[4] Shi W., Jin L., Liu J., Zhang C., Mi Y., Shi J., Wang H., Liang X. (2021). Blastocyst morphology is associated with the incidence of monozygotic twinning in assisted reproductive technology. Am. J. Obstet. Gynecol..

[5] Ma B.X., Zhang H., Jin L., Huang B. (2022). Neonatal Outcomes of Embryos Cultured in a Time-Lapse Incubation System: An Analysis of More Than 15,000 Fresh Transfer Cycles. Reprod. Sci..

[6] Sciorio R., Meseguer M. (2021). Focus on time-lapse analysis: Blastocyst collapse and morphometric assessment as new features of embryo viability. Reprod. Biomed. Online.

[7] Apter S., Ebner T., Freour T., Guns Y., Kovacic B., Le Clef N., Marques M., Meseguer M., Montjean D., ESHRE Working group on Time-lapse technology (2020). Good practice recommendations for the use of time-lapse technology. Hum. Reprod. Open.

[8] Revelli A., Canosa S., Carosso A., Filippini C., Paschero C., Gennarelli G., Piane L.D., Benedetto C. (2019). Impact of the addition of Early Embryo Viability Assessment to morphological evaluation on the accuracy of embryo selection on day 3 or day 5: A retrospective analysis. J. Ovarian Res..

[9] Ebner T., Oppelt P., Radler E., Allerstorfer C., Habelsberger A., Mayer R.B., Shebl O. (2017). Morphokinetics of vitrified and warmed blastocysts predicts implantation potential. J. Assist. Reprod. Genet..

[10] Desai N., Goldberg J.M., Austin C., Falcone T. (2018). Are cleavage anomalies, multinucleation, or specific cell cycle kinetics observed with time-lapse imaging predictive of embryo developmental capacity or ploidy?. Fertil. Steril..

[11] Berntsen J., Rimestad J., Lassen J.T., Tran D., Kragh M.F. (2022). Robust and generalizable embryo selection based on artificial intelligence and time-lapse image sequences. PLoS ONE.

[12] Ueno S., Berntsen J., Ito M., Uchiyama K., Okimura T., Yabuuchi A., Kato K. (2021). Pregnancy prediction performance of an annotation-free embryo scoring system on the basis of deep learning after single vitrified-warmed blastocyst transfer: A single-center large cohort retrospective study. Fertil. Steril..

[13] Tran D., Cooke S., Illingworth P.J., Gardner D.K. (2019). Deep learning as a predictive tool for fetal heart pregnancy following time-lapse incubation and blastocyst transfer. Hum. Reprod..

[14] Ueno S., Berntsen J., Ito M., Okimura T., Kato K. (2022). Correlation between an annotation-free embryo scoring system based on deep learning and live birth/neonatal outcomes after single vitrified-warmed blastocyst transfer: A single-centre, large-cohort retrospective study. J. Assist. Reprod. Genet..

[15] Liu Y., Chapple V., Feenan K., Roberts P., Matson P. (2016). Time-lapse deselection model for human day 3 in vitro fertilization embryos: The combination of qualitative and quantitative measures of embryo growth. Fertil. Steril..

[16] Petersen B.M., Boel M., Montag M., Gardner D.K. (2016). Development of a generally applicable morphokinetic algorithm capable of predicting the implantation potential of embryos transferred on Day 3. Hum. Reprod..

[17] Yang L., Cai S., Zhang S., Kong X., Gu Y., Lu C., Dai J., Gong F., Lu G., Lin G. (2018). Single embryo transfer by Day 3 time-lapse selection versus Day 5 conventional morphological selection: A randomized, open-label, non-inferiority trial. Hum. Reprod..

[18] Meseguer M., Herrero J., Tejera A., Hilligsoe K.M., Ramsing N.B., Remohi J. (2011). The use of morphokinetics as a predictor of embryo implantation. Hum. Reprod..

[19] Tokuoka Y., Yamada T.G., Mashiko D., Ikeda Z., Kobayashi T.J., Yamagata K., Funahashi A. (2022). An explainable deep learning-based algorithm with an attention mechanism for predicting the live birth potential of mouse embryos. Artif. Intell. Med..

[20] Rubio I., Galán A., Larreategui Z., Ayerdi F., Bellver J., Herrero J., Meseguer M. (2014). Clinical validation of embryo culture and selection by morphokinetic analysis: A randomized, controlled trial of the EmbryoScope. Fertil. Steril..

[21] Adamson G.D., Abusief M.E., Palao L., Witmer J., Palao L.M., Gvakharia M. (2016). Improved implantation rates of day 3 embryo transfers with the use of an automated time-lapse-enabled test to aid in embryo selection. Fertil. Steril..

[22] VerMilyea M.D., Tan L., Anthony J.T., Conaghan J., Ivani K., Gvakharia M., Boostanfar R., Baker V.L., Suraj V., Chen A.A. (2014). Computer-automated time-lapse analysis results correlate with embryo implantation and clinical pregnancy: A blinded, multi-centre study. Reprod. Biomed. Online.

[23] Kirkegaard K., Campbell A., Agerholm I., Bentin-Ley U., Gabrielsen A., Kirk J., Sayed S., Ingerslev H.J. (2014). Limitations of a time-lapse blastocyst prediction model: A large multicentre outcome analysis. Reprod. Biomed. Online.

[24] Freour T., Le Fleuter N., Lammers J., Splingart C., Reignier A., Barriere P. (2015). External validation of a time-lapse prediction model. Fertil. Steril..

[25] Ezoe K., Shimazaki K., Miki T., Takahashi T., Tanimura Y., Amagai A., Sawado A., Akaike H., Mogi M., Kaneko S. (2022). Association between a deep learning-based scoring system with morphokinetics and morphological alterations in human embryos. Reprod. Biomed. Online.

[26] Kato K., Ueno S., Berntsen J., Kragh M.F., Okimura T., Kuroda T. (2023). Does embryo categorization by existing artificial intelligence, morphokinetic or morphological embryo selection models correlate with blastocyst euploidy rates?. Reprod. Biomed. Online.

[27] Avery B., Madison V., Greve T. (1991). Sex and development in bovine in-vitro fertilized embryos. Theriogenology.

[28] Mao Y., Zeng M., Meng Y.M., Wang C., Luo Y., Li L. (2023). Effect of blastocyst quality on human sex ratio at birth in a single blastocyst frozen thawed embryo transfer cycle. Gynecol. Endocrinol..

[29] Lou H., Li N., Zhang X., Sun L., Wang X., Hao D., Cui S. (2020). Does the sex ratio of singleton births after frozen single blastocyst transfer differ in relation to blastocyst development?. Reprod. Biol. Endocrinol..

[30] Cai H., Ren W., Wang H., Shi J. (2022). Sex ratio imbalance following blastocyst transfer is associated with ICSI but not with IVF: An analysis of 14,892 single embryo transfer cycles. J. Assist. Reprod. Genet..

[31] Menezo Y.J., Chouteau J., Torello J., Girard A., Veiga A. (1999). Birth weight and sex ratio after transfer at the blastocyst stage in humans. Fertil. Steril..

[32] Ebner T., Tritscher K., Mayer R.B., Oppelt P., Duba H.-C., Maurer M., Schappacher-Tilp G., Petek E., Shebl O. (2016). Quantitative and qualitative trophectoderm grading allows for prediction of live birth and gender. J. Assist. Reprod. Genet..

[33] Gardner D.K., Wale P.L. (2013). Analysis of metabolism to select viable human embryos for transfer. Fertil. Steril..

[34] Epstein C.J., Smith S., Travis B., Tucker G. (1978). Both X chromosomes function before visible X-chromosome inactivation in female mouse embryos. Nature.

[35] Tarin J.J., Garcia-Perez M.A., Hermenegildo C., Cano A. (2014). Changes in sex ratio from fertilization to birth in assisted-reproductive-treatment cycles. Reprod. Biol. Endocrinol..

[36] Tan K., An L., Miao K., Ren L., Hou Z., Tao L., Zhang Z., Wang X., Xia W., Liu J. (2016). Impaired imprinted X chromosome inactivation is responsible for the skewed sex ratio following in vitro fertilization. Proc. Natl. Acad. Sci. USA.

[37] Alfarawati S., Fragouli E., Colls P., Stevens J., Gutiérrez-Mateo C., Schoolcraft W.B., Katz-Jaffe M.G., Wells D. (2011). The relationship between blastocyst morphology, chromosomal abnormality, and embryo gender. Fertil. Steril..

[38] Bronet F., Nogales M.-C., Martínez E., Ariza M., Rubio C., García-Velasco J.-A., Meseguer M. (2015). Is there a relationship between time-lapse parameters and embryo sex?. Fertil. Steril..

