# Supplementary material for: Predictive Ability of an Objective and Time-Saving Blastocyst Scoring Model on Live Birth
Source: Biomedicines. 2025 Jul 15;13(7):1734. doi: 10.3390/biomedicines13071734 (PMC12292090; doi:10.3390/biomedicines13071734)
Supplement: Supplementary file 1 [file biomedicines-13-01734-s001.zip › biomedicines-3702284-supplementary.pdf]

Supplementary Table S1 Perinatal and Neonatal outcomes for monozygotic twins pregnancy in different iDAScore groups

iDAScore group	All	1.0-8.0	8.1-8.9	9.0-9.3	9.4-9.9	*p* value
Live birth, *n*	92	12	16	36	28	/
Gestational age, mean ± SD, weeks	34.6 ± 2.3	35.5 ± 1.9	34.3 ± 3.0	34.3 ± 2.4	34.8 ± 1.9	0.840[a]
Early preterm birth (<37 weeks), n (%)	38 (82.6%)	4 (66.7%)	5 (62.5%)	15 (83.3%)	14 (100%)	0.099[b]
Very early preterm birth (<32 weeks), n (%)	5 (10.9%)	0 (0.0%)	1 (12.5%)	3 (16.7%)	1 (7.1%)	0.664[b]
Types of pregnancy complication, n (%)						0.848[c]
Gestational hypertension, n (%)	1 (2.2%)	1 (8.3%)	0 (0.0%)	0 (0.0%)	0 (0.0%)	
Gestational diabetes, n (%)	0 (0.0%)	0 (0.0%)	0 (0.0%)	0 (0.0%)	0 (0.0%)	
Pre-eclampsia, n (%)	0 (0.0%)	0 (0.0%)	0 (0.0%)	0 (0.0%)	0 (0.0%)	
Placenta previa, n (%)	0 (0.0%)	0 (0.0%)	0 (0.0%)	0 (0.0%)	0 (0.0%)	
Premature rupture of membranes, n (%)	2 (2.2%)	0 (0.0%)	0 (0.0%)	2 (5.6%)	0 (0.0%)	
Birth weight, n (%)						0.541[d]
＜1500 g	8 (8.7%)	0 (0.0%)	4 (25.0%)	2 (5.5%)	2 (7.1%)	
1500-2499 g	50 (54.3%)	8 (66.7%)	6 (37.5%)	20 (55.6%)	16 (57.1%)	
2500-3999 g	34 (37.0%)	4 (33.3%)	6 (37.5%)	14 (38.9%)	10 (35.7%)	
≥ 4000 g	0 (0.0%)	0 (0.0%)	0 (0.0%)	0 (0.0%)	0 (0.0%)	
Infant sex, n (%)						0.228[e]
Male infant	48 (52.2%)	3 (25.0%)	10 (62.5%)	18 (50.0%)	17 (60.7%)	
Female infant	44 (47.8%)	9 (75.0%)	6 (37.5%)	18 (50.0%)	11 (39.3%)	

SD: standard deviation.

[a.] *P* value was calculated by ANOVA test with Bonferroni correction for gestational age.

[b.] *P* value was calculated by Cochrane-Armitage trend test for early preterm birth (<37 weeks) and very early preterm birth (<32 weeks).

[c.] *P* value was calculated by Chi-square analysis for different types of pregnancy complication.

[d] P value was calculated by Chi-square analysis for birth weight.

[e] P value was calculated by Chi-square analysis for infant sex.

Supplementary Table S2 Birth defect in different iDAScore groups

iDAScore group	All	1.0-8.0	8.1-8.9	9.0-9.3	9.4-9.9	*P* value
Live birth, *n*	3014	558	747	1025	684	/
Birth defect rate in infant sex, n (%)						0.841[a]
Birth defect rate in male infant, n (%)	11 (0.4%)	2 (0.4%)	4 (0.5%)	4 (0.4%)	1 (0.1%)	
Birth defect rate in female infant, n (%)	12 (0.4%)	3 (0.5%)	3 (0.4%)	3 (0.3%)	3 (0.4%)	
Birth defect rate in singleton/twin, n (%)						/
Birth defect rate in singleton, n (%)	23 (0.8%)	5 (0.8%)	7 (0.9%)	7 (0.7%)	4 (0.6%)	
Birth defect rate in twins, n (%)	0 (0.0%	0 (0.0%)	0 (0.0%)	0 (0.0%)	0 (0.0%)	
Types of birth defect, n (%)						0.848[b]
Nervous system	1 (0.0%)	0 (0.0%)	0 (0.0%)	1 (0.1%)	0 (0.0%)	
Facial and head and neck	2 (0.0%)	0 (0.0%)	1 (0.1%)	1 (0.1%)	0 (0.0%)	
Cardiovascular system	3 (0.0%)	0 (0.0%)	1 (0.1%)	0 (0.0%)	2 (0.3%)	
Respiratory system	2 (0.0%)	0 (0.0%)	1 (0.1%)	1 (0.1%)	0 (0.0%)	
Digestive system	3 (0.1%)	0 (0.0%)	1 (0.1%)	1 (0.1%)	1 (0.1%)	
Urinary system	7 (0.2%)	3 (0.5%)	2 (0.3%)	1 (0.1%)	1 (0.1%)	
Skeleton and muscles	5 (0.2%)	2 (0.3%)	1 (0.0%)	2 (0.2%)	0 (0.0%)	
Others	0 (0.0%	0 (0.0%)	0 (0.0%)	0 (0.0%)	0 (0.0%)	

[a] P value was calculated by Chi-square analysis for birth defects in male and female infants.

[b] P value was calculated by Chi-square analysis for different types of birth defect.
